# Use of Sentinel-3 OLCI Images and Machine Learning to Assess the Ecological Quality of Italian Coastal Waters

**DOI:** 10.3390/s23229258

**Published:** 2023-11-18

**Authors:** Chiara Lapucci, Andrea Antonini, Emanuele Böhm, Emanuele Organelli, Luca Massi, Alberto Ortolani, Carlo Brandini, Fabio Maselli

**Affiliations:** 1 National Research Council (CNR), Institute of Marine Science (ISMAR), Via Madonna del Piano 10, 50019 Sesto Fiorentino, Florence, Italy; emanuele.bohm@cnr.it (E.B.); carlo.brandini@cnr.it (C.B.); 2 LaMMA Consortium, Via Madonna del Piano 10, 50019 Sesto Fiorentino, Florence, Italy; antonini@lamma.toscana.it (A.A.); alberto.ortolani@cnr.it (A.O.); 3 National Research Council (CNR), Institute of Marine Science (ISMAR), Via Fosso del Cavaliere 100, 00133 Rome, Italy; emanuele.organelli@cnr.it; 4 Dipartimento di Biologia, Università Degli Studi di Firenze, Via Micheli 1, 50121 Florence, Italy; luca.massi@unifi.it; 5 National Research Council (CNR), Institute for BioEconomy (IBE), Via Madonna del Piano 10, 50019 Sesto Fiorentino, Florence, Italy

**Keywords:** TRIX, water quality, satellite, Copernicus Marine Service, random forest

## Abstract

Understanding and monitoring the ecological quality of coastal waters is crucial for preserving marine ecosystems. Eutrophication is one of the major problems affecting the ecological state of coastal marine waters. For this reason, the control of the trophic conditions of aquatic ecosystems is needed for the evaluation of their ecological quality. This study leverages space-based Sentinel-3 Ocean and Land Color Instrument imagery (OLCI) to assess the ecological quality of Mediterranean coastal waters using the Trophic Index (TRIX) key indicator. In particular, we explore the feasibility of coupling remote sensing and machine learning techniques to estimate the TRIX levels in the Ligurian, Tyrrhenian, and Ionian coastal regions of Italy. Our research reveals distinct geographical patterns in TRIX values across the study area, with some regions exhibiting eutrophic conditions near estuaries and others showing oligotrophic characteristics. We employ the Random Forest Regression algorithm, optimizing calibration parameters to predict TRIX levels. Feature importance analysis highlights the significance of latitude, longitude, and specific spectral bands in TRIX prediction. A final statistical assessment validates our model’s performance, demonstrating a moderate level of error (MAE of 0.51) and explanatory power (R^2^ of 0.37). These results highlight the potential of Sentinel-3 OLCI imagery in assessing ecological quality, contributing to our understanding of coastal water ecology. They also underscore the importance of merging remote sensing and machine learning in environmental monitoring and management. Future research should refine methodologies and expand datasets to enhance TRIX monitoring capabilities from space.

## 1. Introduction

Coastal ecosystems are vital components of our environment, providing numerous ecological, economic, and societal benefits. Water ecological quality refers to the health and sustainability of aquatic ecosystems. It underscores the interplay of biodiversity, nutrient cycling, and trophic dynamics, which collectively shape the overall health of aquatic ecosystems [1]. Ecological quality draws from ecosystem theory, emphasizing that these are interconnected systems where changes in one component can have cascading effects throughout, highlighting the importance of balance and stability for sustainability [2].

Water ecological quality is, therefore, a fundamental concept within the European Union (EU) environmental framework, standing at the heart of environmental policies and regulations, with a focus on ensuring the health and sustainability of ecosystems. In particular, the EU Water Framework Directive (WFD, EU, 2000) [3] (WFD) plays a central role in assessing water ecological quality, defining ecological status as a measure of the structure and functioning of coastal ecosystems. Both the Water Framework Directive (WFD) and the Marine Strategy Framework Directive (MSFD, EU, 2008) [4] adopt a holistic approach designed to safeguard and preserve the marine environment.

A plethora of parameters and tools are utilized to assess the health and functioning of coastal ecosystems [5]. The conventional methods of evaluating water quality rely on the in situ monitoring of a wide range of water quality parameters, such as nutrient levels, temperature, salinity, pH, oxygen levels, and pollutant concentrations. In fact, both the WFD and MSFD mainly rely on in situ monitoring [6]. The data collected are used to estimate indicators, including the Trophic Level Index (TLI) [7], Coastal Eutrophication Potential (CEP) [8], Ocean Health Index (OHI) [9], and Ecological Quality Ratios (EQRs) [10], which are important for assessing the ecological quality of coastal waters, aiding in the evaluation of various ecosystem aspects, including nutrient dynamics, trophic interactions, biodiversity, and pollution. In particular, the Trophic Index (TRIX) [11] is a quantitative index specifically developed to assess the trophic status of seawater. It combines factors that describe phytoplankton biomass, nutrient availability, and oxygen as indicators of environmental disturbance in the water quality, resulting in a numerical index based on the Chl-a concentration, oxygen absolute deviation from saturation, total phosphorus, nitrogen concentration, and water clarity. The advantage of using TRIX over CEP, TLI, OHI, and EQR for ecological quality assessment in coastal waters lies in its simplicity, wide applicability, and established framework.

The in situ monitoring of water quality is improving, for example, with the recent developments of instruments like multiparametric probes [12]. While it provides precise measurements, however, in situ monitoring does not afford synoptic views and is constrained by temporal gaps, high operational costs, and labor-intensive data collection. These issues can be partially addressed using Earth Observation (EO) techniques, which offer a number of advantages, including improved spatial and temporal coverage, frequent data acquisition, reduced costs, and the ability to access remote areas [13]. The advent of EO techniques, particularly from satellite platforms, has, therefore, revolutionized water quality assessment and regulatory compliance. The next section provides a brief overview of this subject, followed by a presentation of the current research objectives.

### Related Work and Research Objectives

EO techniques employ the relationships between water optical properties and surface concentrations of parameters linked to ecological quality, such as Chl-a, Suspended Particulate Matter (SPM), turbidity, Colored Dissolved Organic Matter (CDOM), etc. [14]. At present, the relationships between spectral data and ecological water quality indices have been only partially investigated in very few works [15,16]. As an example, satellite-derived surface Chl-a concentration is widely used as a proxy of the prevalent trophic status at a given oceanic location [17]. The main environmental conditions of marine coastal areas are, in fact, strongly related to phytoplankton abundance, biomass, and dynamics and can be estimated from the concentration of Chl-a. Other water quality parameters are also detectable by remote sensing techniques, including SPM, CDOM, turbidity, and sea surface temperature (SST). These data can be used to understand the health of coastal ecosystems, track changes over time, and inform management decisions.

Satellite remote sensing techniques, in particular, can provide information on coastal water quality over large areas and at high frequencies, which is difficult to achieve with in situ sampling alone [18]. This can be very useful for monitoring coastal and lake water quality changes over time or for identifying areas of concern that may require further sampling [19,20,21,22,23,24,25]. As for coastal water quality monitoring, satellites are used for water quality studies connected to Harmful Algal Blooms [26]. Many satellites dedicated to ocean color observations have been active since the late 1970s, and spatial and spectral resolution have been improved with technological advances with respect to the former generation of satellites. The current multispectral sensors onboard satellites will be further improved by the introduction of hyperspectral sensors that will potentially allow more advanced water quality monitoring of the coastal zones.

These technological advancements could be particularly relevant for the estimation of an index like the TRIX, which is intrinsically dependent on the concentration of a major optically active seawater constituent, Chl-a. As the TRIX is normally computed by in situ data, with all the limits connected, in fact, the exciting possibility of obtaining it from a satellite ushers the potential to monitor, simultaneously, large sea areas with high temporal frequency. The exploration of this possibility is based on the pioneering work of Massi et al. (2019) [27], which showed how hyperspectral observations taken in situ are informative on the TRIX levels of port waters. Pieri et al. (2021) [28] confirmed this possibility using high spatial resolution multispectral imagery taken from satellite platforms. A further study [29] extended the analysis to the use of multispectral observations taken by the Sentinel 3 mission through the Ocean and Land Color Instrument (OLCI) sensor, which is particularly suitable for monitoring medium-scale seawater phenomena. This last study preliminarily explored the possibility of estimating the TRIX in the coastal areas of Tuscany (Central Italy) by applying standard regression methods to Remote Sensing Reflectance (Rrs) and standard Level 2 Sentinel 3 OLCI Full Resolution (FR) data (Chl-a concentration, suspended sediment concentration, and diffuse attenuation coefficient of light at 490 nm).

These investigations suggest the feasibility of leveraging direct and indirect relationships between the TRIX and optically active seawater components to establish an alternative monitoring approach based on satellite observations. The same studies, however, also pointed out important issues needing further scientific and technological advancement. First, an optimal characterization of seawater TRIX values should be based on detailed spectral information, which can be currently obtained only from last-generation multispectral sensors. Second, the complex and irregular relationships between in situ TRIX values and spectral signatures should be addressed with the use of extremely efficient and flexible nonparametric estimation algorithms.

Building on these premises, the current study endeavors to establish a remote sensing-based approach for monitoring and mapping the ecological conditions of marine coastal waters through the use of the TRIX. The achievement of this objective requires addressing the two mentioned issues, which is carried out by the application of advanced data processing methods to Sentinel 3 OLCI imagery. In particular, the study explores the application of machine learning techniques to OLCI and in situ data to obtain information about the TRIX levels of Italian coastal waters. In this way, our approach addresses the complex nonlinear relationships existing between spectral and in situ observations related to the TRIX.

The main novelty of the paper lies in the synergistic combination of data from state-of-the-art ocean color satellite sensors and machine learning to obtain information on the ecological quality of coastal waters. The scientific objectives of the paper are:

1. The exploitation of the latest generation high-resolution (300 m) OLCI images applied to TRIX estimation, highlighting the potential for water quality assessment as well as possible broader environmental applications.
2. The pioneering use of machine learning algorithms to provide a novel framework for assessing ecological quality in coastal waters.
3. A methodological advancement towards dealing with the complexities of coastal environments, overcoming the limitations of in situ monitoring and enabling more effective environmental management.
4. Enhancing the versatility of the proposed methodology for application in coastal waters, where multiple environmental factors and stressors interact.

The paper is organized as follows. First, the TRIX is introduced, followed by a description of the study area and the dataset used: OLCI FR data and environmental parameters for TRIX estimation. The preprocessing and processing steps applied to in situ and satellite data are then illustrated, with a specific focus on the machine learning algorithm used. Section 3 reports on the performance of this algorithm for TRIX prediction, together with the TRIX distribution in the study area. The final section discusses these results and provides insights for possible future investigations.

## 2. Materials and Methods

The TRIX index, a key indicator of trophic condition, is described (Section 2.1). Then, the peculiar characteristics of the study area (Section 2.2) and the database of in situ data are presented (Section 2.3). The use of Sentinel 3 OLCI satellite data (Section 2.4) and the application of machine learning techniques for data processing (Section 2.5) are detailed. An emphasis is placed on the essential data preprocessing steps, including pre-filtering, outlier detection, and normalization (Sections Pre-Filtering and Outlier Detection and Selection). The training and application of the Random Forest Regressor (RFR) algorithm (Section Training and Application of RFR) to retrieve the TRIX values is described. These methodological details provide a foundation for the subsequent investigation regarding the trophic conditions of the coastal waters of the Ligurian, Tyrrhenian, and Ionian Seas.

### 2.1. TRIX

The TRIX [11] is an established metric for evaluating trophic conditions within aquatic ecosystems. The TRIX is, in fact, integrated into the Italian law for coastal marine environment status [30] implementing the EU-WFD, and the environmental attributes used to estimate it are also monitored by the EU-MSFD [31] to characterize the trophic status of all European coastal waters.

The TRIX is useful to classify water bodies and is built on their nutrient enrichment and primary productivity, thereby distinguishing oligotrophic (low nutrient levels) from eutrophic (high nutrient levels) states; oxygen is included to indicate the ecosystem’s ability to support life at different trophic levels. The basis of this assessment lies in the trophic hierarchy, which includes primary producers like phytoplankton, primary consumers like zooplankton, and higher trophic levels, including fishes. The TRIX is typically defined through a linear combination of the logarithms of four environmental factors directly linked to productivity. These factors are the Chl-a concentration in µg/L, which indicates phytoplankton biomass; the absolute value of the percentage deviation from oxygen saturation (aDO%); and the total nitrogen (N) and total phosphorus (P) dissolved in water, which are both measured in µg/L.

Consequently, the TRIX can be calculated using the following Equation (1):

(1) $$\frac{TRIX=\left[log\;\left(\;Chl-a\;\times \left|aDO\%\right|\times N\times P\right)-\left(-1.5\right)\right]}{1.2}$$

The scaling constants 1.5 and 1.2 in Equation (1) are derived from an extended dataset used to assess long-term trends and spatial trophic patterns in European coastal waters [32]. The TRIX score ranges from 0 to 10, classifying four trophic conditions: 0–4 corresponds to high quality and low trophic status, termed “High”; 4–5 represents good quality and moderate trophic status, labeled “Good”; 5–6 indicates moderate quality and high trophic status, referred to as “Moderate”; and 6–10 signifies degraded quality and very high trophic status, categorized as “Poor”.

Although first designed for lakes, the TRIX has been extended to coastal waters, initially for eutrophication assessment. In coastal environments, the TRIX assumes the role of assessing the effects of nutrient enrichment stemming from anthropogenic activities, such as agricultural practices and urban runoff.

### 2.2. Study Area

**Figure 1:**
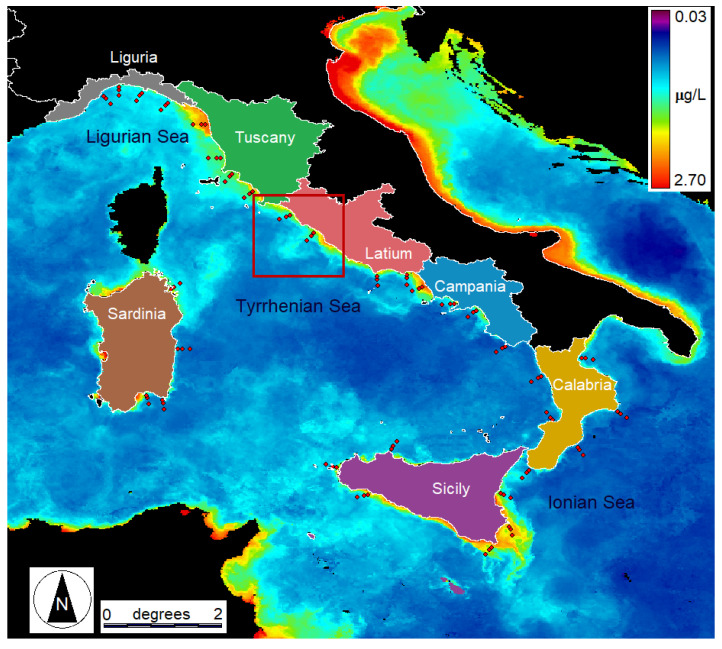
ISPRA EU-MSFD monitoring stations in the study area (red dots) are superimposed onto OLCI FR Chl-a map (10–17 February 2017, average of daily images). The red box in coastal central Italy indicates the position of the illustrative TRIX map shown in the Section 3.

The coastal waters of the Ligurian, Tyrrhenian, and Ionian (around Sicily) Seas show distinct geographical, physical, biogeochemical, trophic, and optical characteristics that reflect their unique marine environments (Figure 1). Geographically, The Ligurian Sea is an arm of the Mediterranean Sea that borders the Italian Riviera, the French Riviera, and the island of Corsica, featuring deep waters. In contrast, the Tyrrhenian Sea presents a broader expanse, surrounded by the Italian mainland, Sardinia, and Sicily. The Sicilian Sea includes the island of Sicily, connecting the Tyrrhenian and Ionian Seas.

The coastal Ligurian Sea exhibits oligo-mesotrophic characteristics, which are primarily attributed to limited nutrient availability. These characteristics are the result of restricted nutrient inputs from internal areas and the influence of the Ligurian Current that, together with the geographical features of the region, hinder the transport of essential nutrients from land to sea.

The trophic diversity of the coastal areas of the Tyrrhenian Sea results from physical and geographical characteristics that include the large estuary of the Tiber River. Along its coastlines, the Tyrrhenian Sea displays varying trophic states, encompassing oligotrophic to mesotrophic conditions. The coastal zone of the Tyrrhenian Sea is characterized by its proximity to the Italian peninsula, Sardinia, and Sicily, each of which contributes unique nutrient inputs to the sea. Additionally, the intricate interplay of multiple oceanic currents, including the inflow of Modified Atlantic Waters, shapes the physical dynamics of the coastal Tyrrhenian Sea. These currents play a pivotal role in determining nutrient availability and distribution, thereby exerting a direct impact on the trophic conditions observed in different coastal regions.

The Ionian Sea exhibits distinct trophic conditions, which are shaped by a combination of geographical and physical factors specific to this region. The waters surrounding Sicily are characterized by their central location in the Mediterranean, diverse geography, and range of trophic conditions that sustain a dynamic marine ecosystem.

### 2.3. Sampling Stations and In Situ Data

The in situ data for the TRIX estimation were obtained from the Italian Institute for Environmental Protection and Research (ISPRA) in the framework of the monitoring activities established by the EU-MSFD (Figure 1). Specifically, the data are distributed by the Centralized Information System (Sistema Informativo Centralizzato, SIC), which collects, manages, and shares the data coming from the monitoring programs within the framework of the EU-MSFD at the community level (http://www.db-strategiamarina.isprambiente.it/, accessed on 2 May 2023). The map includes Chl-a, a level 2 product obtained from OLCI FR via the OC4ME algorithm.

The EU-MSFD Module 1 is dedicated to the monitoring of the chemical–physical parameters of the water column, pelagic habitats, and the contaminants present in the water, including the ones used to estimate the TRIX. The areas are chosen along a coast-to-offshore transect in front of some of the stations identified for the implementation of the EU-WFD because they are characterized by different sources of pollution, both natural and anthropogenic: river mouths and/or nutrient inputs, upwelling (presence of upwelling areas of deep waters usually rich in nutrients) or downwelling (areas of accumulation and sinking of high-density and low-temperature water below lower-density and higher-temperature waters), and port areas. In this study, we selected all the monitoring points of the EU-MSFD located in the Ligurian and Tyrrhenian Seas. The transects include monitoring stations at a distance of 3, 6, and 12 NM from the coast for which OLCI Full Resolution (FR) data are available.

For each sampling site, four environmental attributes were used to compute the TRIX: Chl-a, aDO%, total N, and total P. To estimate the TRIX using machine learning, the following environmental parameters of the ISPRA database were also included in the input dataset for machine learning: latitude, longitude, distance from the coast (ClosestCoast), sea depth, and mixing characteristics of the water column (Mixing) [4].

### 2.4. Sentinel 3 OLCI Satellite Data

OLCI is a high-resolution, multispectral imaging spectrometer onboard the Sentinel-3A and Sentinel-3B satellites. It provides 21 spectral bands in the visible and near-infrared (VNIR) range, with a spatial resolution of 300 meters at nadir. This makes OLCI a valuable tool for monitoring the coastal zone color features.

OLCI scans the Earth’s surface using a push-broom method. The spatial resolution of OLCI FR is approximately 300 m at a sub-satellite point [33]. Sentinel 3-A and 3-B form a near-polar orbit satellite constellation: 3-A was launched in 2016 and 3-B in 2018. At the latitudes of interest, the combination of the OLCI A and B scenes provides almost daily coverage, while 2 days are required at the equator.

In this work, we used the Sentinel 3 OLCI FR products (https://doi.org/10.48670/moi-00299, accessed on 2 May 2023) provided by Copernicus Marine Service. Each OLCI L2 granule, before being merged with the others taken from the same sensor and from the same day, is quality checked via the application of the L2 flags provided by the European Space Agency (ESA) (https://sentinels.copernicus.eu/web/sentinel/technical-guides/sentinel-3-olci/level-2/quality-and-science-flags-op, accessed on 2 May 2023). All 10 sensor bands’ directional surface water reflectances, centered at 400, 412.5, 442.5, 490, 510, 560, 620, 665, 673.75, and 681.25 nanometers in the visible portion of the spectrum, were considered. Chl-a, in particular, an important component of the in situ TRIX estimation, is typically observed in spectral regions that are sensitive to the molecular absorption and scattering features: Band Ratios (BR) of green (510 nm) and blue wavelengths (442.5, 490 nm) are used here as a proxy for Chl-a concentrations [33]. To minimize the interference of other factors, such as the color of the water, a maximum band ratio (MBR) approach is employed. The MBR selects the most suitable spectral bands within the absorption range of Chl-a, ensuring accurate estimates while mitigating potential errors [34].

The images collected within 1 day of the in situ matchup sampling were processed using the ESA Sentinel Application Platform (SNAP) v8.0 and -Sentinel-3 Toolbox (S3TBX) v8.0.6 (http://step.esa.int, accessed on 2 May 2023). The matchup pixel extraction was performed via SNAP PixEx operator 1.3 (Copyright (C) 2011 Brockmann Consult GmbH (info@brockmann-consult.de) on a 3 × 3 pixel window and averaged, via a dedicated procedure in Python. The investigation then focused on 105 OLCI scenes, from which the values of standard Level 2 products were extracted in correspondence with 340 in situ TRIX observations.

### 2.5. Data Processing

Machine learning can be used to analyze large and complex water quality datasets to make predictions, detect patterns, classify water bodies, optimize monitoring programs, and develop early warning systems, spatial maps, and time series analyses [35]. It can also be used to integrate with remote sensing data to improve the spatial and temporal coverage, accuracy, and precision of coastal water quality data. This can help to reduce the costs and time associated with data analysis, target further sampling and monitoring efforts, and develop management strategies to address water quality problems.

There are at least four knowledge gaps in the current use of satellite-derived data and in situ sampling with a machine learning approach to understand coastal water quality: a lack of standardized methods and protocols; the limited availability of data; a need for more interpretable models; and a lack of operationalization. In the present work, we address the first and third gaps by using the RFR. It is a supervised machine learning algorithm that is widely used in regression techniques. This algorithm, in fact, is accurate, interpretable, robust to outliers, and flexible in terms of the range of parameters that can be predicted. The RFR supervised algorithm uses an ensemble method to make predictions based on various decision trees, each of them producing a specific prediction. It outperforms other machine learning algorithms in terms of accuracy, robustness, ability to handle complex relationships, interpretability, and scalability. RFR, in fact, is able to handle nonlinear relationships between the input and output variables because it uses decision trees. This is important for environmental studies, which involve complex relationships between land surface attributes and ecological indicators.

Owing to these properties, RFR has been shown to be well-suited to assessing the ecological quality of seawater using remotely sensed data [36]. RFR is, therefore, an optimal candidate for assessing the ecological quality of seawater using Sentinel-3 OLCI images. Some studies report, in fact, the use of RFR for water quality assessment from satellite imagery, albeit not specifically from the OLCI sensor [37,38].

#### 2.5.1. Preprocessing of In Situ Data

Before applying the RFR algorithm, several data preprocessing steps were applied to the in situ observations, such as pre-filtering, outlier detection, and data normalization. The above steps were essential to ensure that the data are in a format suitable for processing by a machine learning algorithm and that the predictors are relevant to the target variable. In fact, data quality factors, such as consistency, completeness, and accuracy, are definitely essential to successful machine learning applications [39], especially in the present case of not very extensive datasets.

##### Pre-Filtering

Data related to nutrients and Chl-a need an accurate initial screening of the raw analytical data [40], usually involving the adoption of the empirical approach recommended by the OECD [41], namely, a base 10 logarithmic transformation was applied, the data points that differed from the mean by more than 2.5 standard deviations (STDs) were excluded, and, subsequently, the new mean values and their corresponding STDs were recalculated. This method effectively removes only a small number of anomalous data points, maintaining the resulting distribution within a variation range of 2 to 3 logarithmic units, which aligns well with the theoretically expected range of the mean ± 2.5 STDs. For Chl-a data distributions, the logarithmic transformation yields normalization and the stabilization of variance, where the STD remains relatively constant, ranging between 0.3 and 0.4 [42].

##### Outlier Detection and Selection

In the process of identifying outliers within the dataset, two key methods were employed. Firstly, a visual analysis using box plots was conducted to provide a graphical representation of the data, aiding in the detection of potential outliers. Additionally, the Interquartile Range method (IQR) was applied to systematically identify outliers in the dataset [43]. It is calculated as the difference between the third quartile (Q3) and the first quartile (Q1); any data point that is more than 1.5 times the IQR below Q1 or above Q3 is considered an outlier. This is a simple, effective, and robust method to automate this outlier detection process, which was implemented through a Python code function.

It is important to note that not all the data points identified as outliers in the box plots were removed. Some of these outliers were retained because they fell within the expected data range before the dataset was narrowed down based on satellite matchup criteria. This methodical approach enhances the data quality and reliability, while also considering specific criteria related to the satellite matchup.

##### Training and Application of RFR

The RFR was applied using the Python Scikit-Learn library (sklearn) [44,45,46] to a set of 17 features, which included data from the Sentinel-3 OLCI Rrs observations centered at 400, 412.5, 442.5, 490, 510, 560, 620, 665, 673.75, 681.25 nm, BR, and MBR, and the following data from the in situ database of the ISPRA: latitude, longitude, closest coast, sea depth, and mixing. The RFR supervised algorithm uses an ensemble method to make predictions, based on various decision trees, each of them producing a specific prediction. The randomization process is in both the sampling of the training dataset to build a tree and the subsetting of features to calculate the output for splitting nodes. The mean prediction of the whole set of trees is the output of the regression.

**Figure 2:**
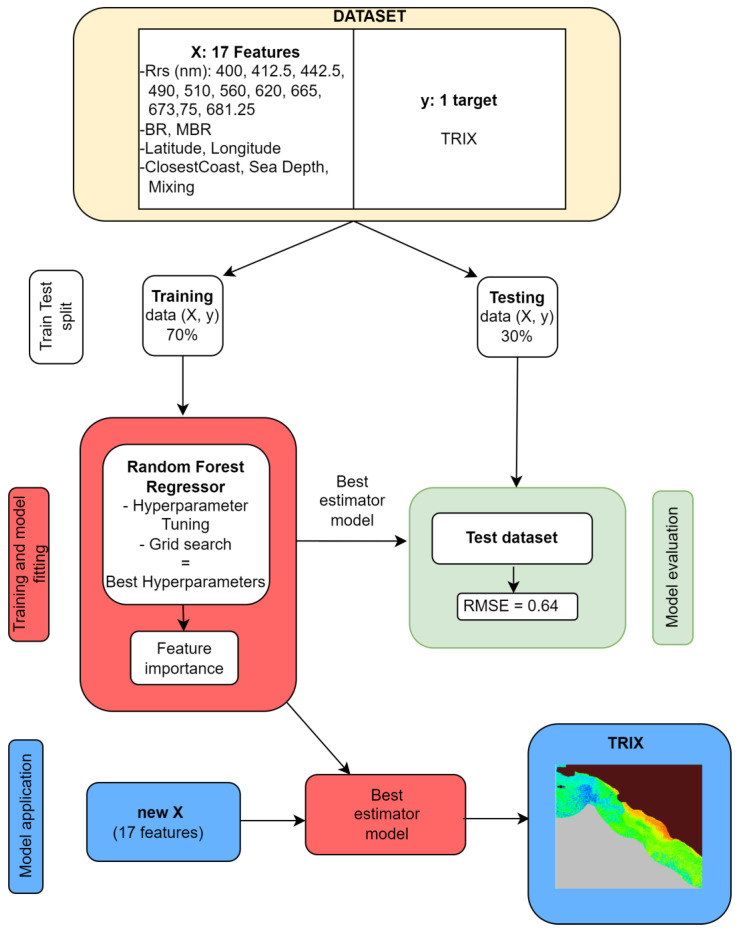
Flow chart of RFR data processing; the image on the bottom right corner shows an example of obtained TRIX map.

Figure 2 shows a flowchart that graphically illustrates the main processing steps involved in the current application of the RFR algorithm. The whole dataset comprising the data points selected by data cleaning, each with 17 independent variables (*X*, features) and one dependent variable (y, target), was subjected to design, calibrate, and evaluate the RFR predictive model. The data underwent meticulous preprocessing using sklearn’s extensive tools for data scaling, normalization, and organization into training and testing subsets. A critical step in the model optimization involved hyperparameter tuning, which was carried out through grid search and cross-validation techniques. In particular, grid search is a hyperparameter optimization technique used in machine learning. It systematically explores a predefined set of hyperparameter values to find the optimal configuration for a model. This process allows one to efficiently tune the model performance and select the best hyperparameters to achieve the highest predictive accuracy. Cross-validation techniques are used in machine learning to assess the performance and generalizability of a predictive model.

Specifically, the hyperparameters to be set for the application of the RFR method were: (1) the number of trees to be used (*n*_estimator); (2) the maximum number of node levels (max_depth) from the root to the leaf of each tree; and (3) the maximum number of features (max_features) to be used as a random subset of the original parameters in the best-split construction of the trees. In order to find the optimal configuration of these parameters, the dataset underwent random splitting into training (70%) and testing (30%) subsets for model fitting and model test phases, respectively. The training data were used for both the model setup, including the definition of the set of parameters, and for the training process of the model. In particular, the best set of parameters was obtained by minimizing the Mean Square Error of the resulting output over the training dataset.

The model was trained with the best hyperparameters on the training data, using a fit method, directly provided by sklearn. The testing set was used for accuracy assessment, which was summarized by means of standard statistics, i.e., the coefficient of determination (R^2^), the Root Mean Square Error (RMSE), and the Mean Absolute Error (MAE).

Feature importance analysis, a key aspect of model interpretation, was also performed, enabling the identification of the relative significance of the independent variables, and aiding in an understanding of their impact on the predictions. The comprehensive integration of sklearn into the research methodology exemplifies its role in data preprocessing, model selection, hyperparameter tuning, and performance evaluation, thereby offering a robust framework for predictive modeling.

Finally, the best estimator model was used to produce a map of the TRIX using OLCI satellite data and ancillary data as a sample of 17 features constituting the *X* input.

## 3. Results

The results section first (Section 3.1) presents a statistical analysis of the key seawater parameters, shedding light on their characteristics across the study area and how they contribute to the observed TRIX variability. In Section 3.2, the regional variations in the TRIX distribution across administrative regions are also shown. The RFR estimates and the hyperparameters employed in the algorithm are shown, as well as the importance of feature analysis. Finally, a discussion of the model’s predictive performance and its significance in contributing to the comprehension of coastal water ecology dynamics is presented.

### 3.1. In Situ Observations

The analysis of the in situ observations reveals that most of the study waters are characterized by relatively low values of the four parameters contributing to the TRIX computation. The average values of Chl-a, aDO%, N, and P are characteristic of coastal waters ranging from oligotrophic to mesotrophic (Table 1). The maximum values of these parameters are usually found in springtime near the outlets of the main rivers.

The four parameters contribute differently to the observed TRIX variability; while the logarithm of Chl-a accounts for about 37% of the total TRIX variation, the other parameters account for smaller percentages (15–32%).

**Figure 3:**
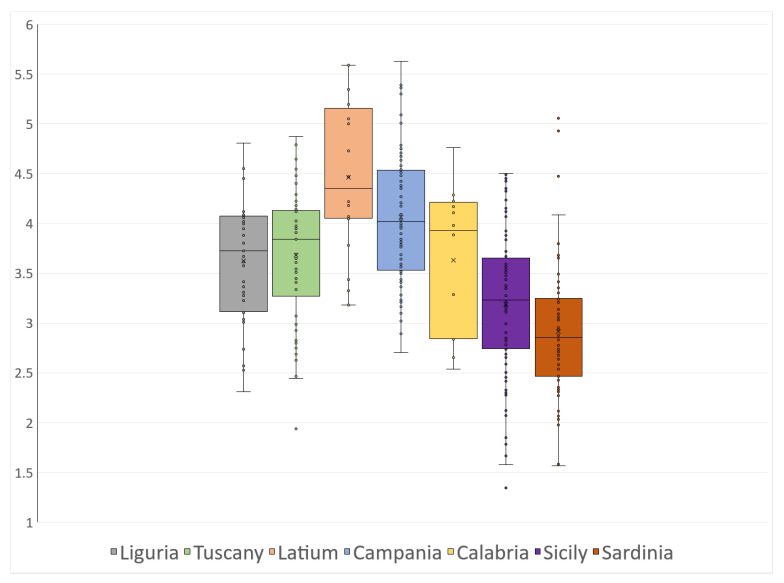
In situ TRIX distribution across the study area. Data are grouped according to the seven administrative regions, which are referred to using the same colors as in Figure 1. The box plot shows the median value of each region and the respective first and third quartiles, minimum and maximum; the black dots are the data points.

The TRIX distribution obtained from in situ measurements grouped in the seven administrative regions reveals distinct geographic patterns along the Italian coasts (Figure 3). A notable maximum is observed near the center of the study area, specifically in the central Tyrrhenian Sea (Latium), while minima are evident in close proximity to the two main islands, Sicily and Sardinia, within the Ionian and central-southern Tyrrhenian Seas. Intermediate TRIX values are found in the Ligurian Sea and the southern Tyrrhenian Sea. Eutrophic conditions prevail in the coastal area near the Tiber estuary in Latium, while Sardinia’s coastal waters show a typical oligotrophic nature.

### 3.2. RFR Estimates

The best set of parameters resulting from the RFR calibration is shown in bold in Table 2. The number of estimators, *n_*estimator (i.e. the number of trees of the forest), chosen is 100, coinciding with the default number of estimators of the software module. A value of 10 was chosen for the depth of the trees, max_depth, allowing the trees to capture complex data relationships without overfitting, resulting in a computationally efficient algorithm. The maximum number of features considered when a split decision is made, max_features, was tested up to 20 and set to 15, influencing the model comprehensiveness and training speed and preventing overfitting as well.

Careful hyperparameter tuning ensured optimal performance of the RFR algorithm, effectively predicting the TRIX levels while preventing overfitting. When evaluated versus the training set, the calibrated RFR algorithm yields an R^2^ of 0.91, an MAE of 0.19, and an RMSE of 0.24.

**Figure 4:**
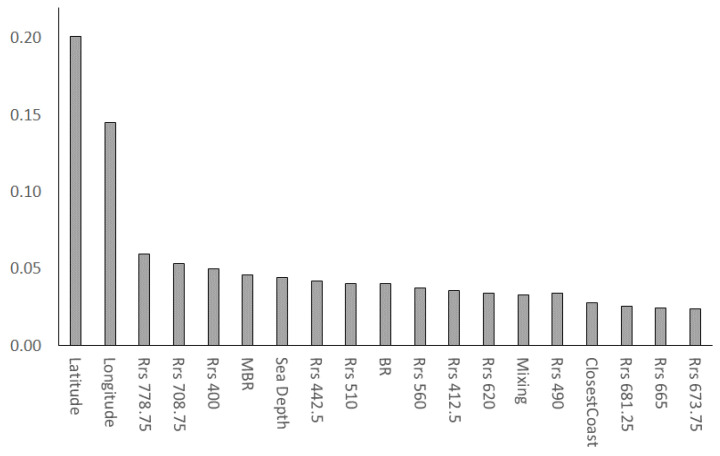
Feature importance histogram.

In Figure 4, we present a feature importance histogram, a vital component of our RFR algorithm analysis. Feature importance quantifies the contribution of each input feature to the model’s predictive performance. In the set of feature importance, the highest score is 0.20, which is relatively high compared to the others. The majority of the scores fall between 0.04 and 0.01, with both the average value and median being equal to 0.03. A smaller group of higher scores falls between 0.2 and 0.05, with an average of 0.09 and a median of 0.06. The feature with a score of 0.20 is relatively more important than the others and likely has a more significant impact on the model predictions. Features with a score between 0.05 and 0.02 are moderately important, but their impact is not as pronounced as the top feature. Features with a score between 0.04 and 0.01 are relatively less important in comparison to the others. A threshold level of 0.05 selects the parameters strongly connected with the TRIX for the dataset and model of the present study.

The relevance of the geographic patterns observed earlier, and the satellite bands used for Chl-a retrieval, is confirmed by the results of the RFR algorithm’s feature importance analysis (Figure 4). Latitude and longitude are identified as the most informative features for the algorithm, with importance scores above a threshold of 0.05. Features such as Rrs 778, Rrs 708, Rrs 400 nm, and MBR (maximum band ratio blue/green) also exhibit importance above the 0.05 threshold, indicating their significance in the model’s predictions. The threshold level of 0.05, as explained above, serves as a criterion to select parameters strongly connected with the TRIX. Features above this threshold are considered highly influential in determining the TRIX values. It ensures that our model primarily relies on the most meaningful variables, optimizing the prediction accuracy while reducing the noise and overfitting. This rigorous feature selection process enhances the robustness of our TRIX predictions.

**Figure 5:**
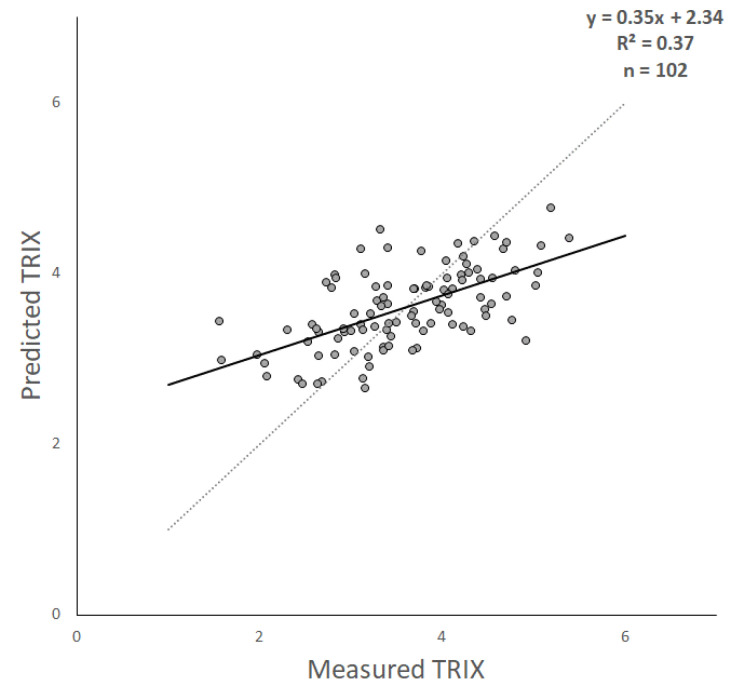
Scatter plot of measured and predicted TRIX. The best fit (solid) and 1:1 (dotted) lines are superimposed on the data points.

The assessment of the RFR TRIX estimates versus the test observations indicates the moderate efficiency of the algorithm (Figure 5). As can be seen in this scatter plot, the TRIX estimates obtained seem to be linearly related to the in situ measurements, with only a minor dispersion around the 1:1 line. The RFR estimates, however, show a reduced TRIX range with respect to the measurements. Table 3 summarizes the key statistics that provide insights into our model’s performance. An MAE of 0.51 indicates a moderate level of error in our predictions, signifying that, on average, the predicted TRIX values deviate by approximately 0.51 units from the observed values. The RMSE of 0.64 quantifies the average squared differences between the predicted and observed TRIX values. Finally, the coefficient of determination (R^2^) of 0.37 indicates a moderate and statistically significant level of explanatory power.

In summary, our model provides valuable insights into TRIX levels in coastal waters while exhibiting a moderate level of error. The scatter plot and statistical analysis validate the model’s performance and contribute to our understanding of complex coastal water ecology dynamics.

**Figure 6:**
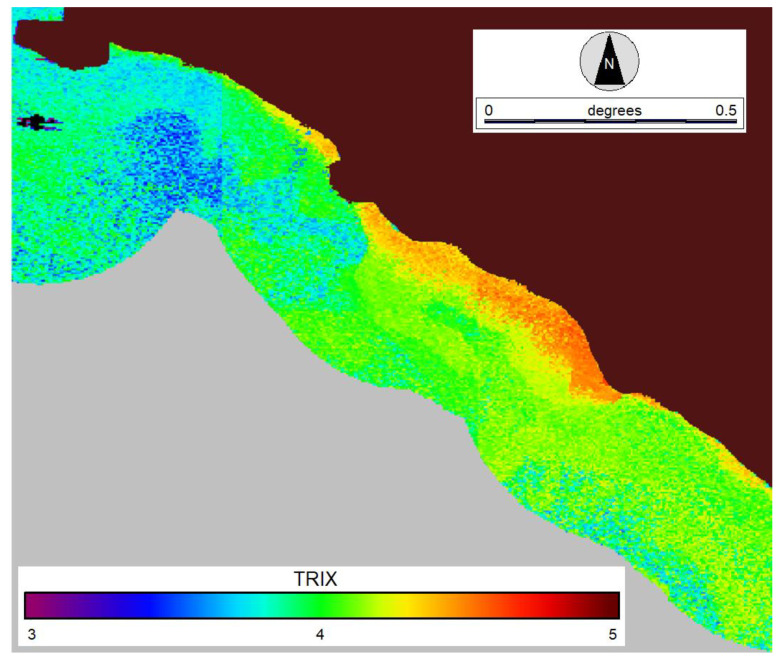
TRIX map obtained for the coastal zone of Latium shown in Figure 1 on 17 March 2017; in grey is the offshore area beyond 0.25° from the coastline.

An example of the TRIX maps, which can be obtained through the described procedure, is displayed in Figure 6 for the spring period of 2017. The map covers the coastal area of Latium outlined in Figure 1 and includes the Tiber River estuary (central part). As expected, the TRIX generally decreases with increasing distance from the coast due to the more oligotrophic nature of the open sea waters. The highest TRIX values are estimated in the Tiber plume, where the seawater is presumably richer in all the basic components of the index.

## 4. Discussion

### 4.1. Remote Sensing of Water Ecological Quality

Many satellites dedicated to ocean color have been active since the late seventies, and the technology has made strong improvements in terms of data quality, such as spatial resolution, in recent times. The current satellite sensors show that characteristics such as spatial resolution have improved with respect to the former generation of satellites, which increases the data quality, and, as a consequence, water quality monitoring. The following water quality parameters are detectable by satellite remote sensing: Chl-a, CDOM, SPM, turbidity, and sea surface temperature (SST). These data can be used to understand the health of coastal ecosystems, track changes over time, and inform management decisions.

With particular regard to water trophic conditions, previous investigations have indicated that high spatial resolution multispectral satellite imagery is informative on the TRIX levels of port and coastal seawaters [25,26,27]. This is primarily based on the direct relationship between the Rrs multispectral signatures of these waters and the respective concentration of Chl-a, which is a major component of the TRIX. However, such a relationship becomes particularly complex and irregular in Case 2 waters, which are characteristic of the coastal zones under investigation. The spectral analysis of these waters is, in fact, a complex challenge that has been the subject of several investigations [47,48].

In addition to the direct link with Chl-a, there is potential for multispectral data to contribute to the estimation of the TRIX through indirect relationships with other TRIX components, such as nitrogen and phosphorus. These components are influenced by the concentrations of other optically active seawater constituents, namely SPM and CDOM. Unfortunately, these relationships are also complex and variable, which makes the estimation of the TRIX particularly challenging.

### 4.2. Current Research Strategy

The approach currently applied to address such challenges is based on two major pillars. The first involves the use of multispectral imagery retrieved by an advanced sensor, the state-of-the-art Sentinel-3 OLCI, which is capable of accurately characterizing the optical properties of coastal seawater at a 300 m resolution. Owing to this property, the data taken by this sensor have been successfully used in several investigations. Su et al. [49], for example, showed the value of OLCI imagery for the prediction of Chl-a concentrations in optically complex Chinese waters.

The second pillar centers on the application of an extremely flexible machine learning algorithm, the RFR. As previously noted, machine learning techniques can be used to combine in situ sampling data with satellite remotely sensed data to create more accurate and precise estimates of coastal water quality parameters. This is because machine learning algorithms can learn the complex relationships between different water quality parameters and the various factors that influence them. Among machine learning algorithms, the RFR demonstrates exceptional capability in adapting to complex and variable data patterns [48]. Extensive studies have, therefore, consistently shown that the RFR can effectively manage the complexity associated with the aforementioned relationships.

### 4.3. RFR Algorithm Applied

The successful implementation of the current research strategy has required the collection and meticulous preprocessing of an extensive dataset, including in situ TRIX observations and OLCI images representative of the inhomogeneous coastal waters within the study area. A preliminary analysis of the in situ observations has provided relevant information on the trophic status of the area. Most of these observations, in fact, denote good to high ecological status; all the recorded TRIX values fall within concentration reference limits denoting ‘sufficient’ ecological status [50]. These findings highlight the geographic variations in trophic conditions across the study regions and underscore their adherence to ecological reference standards, which is indicative of their overall health and functioning.

The developed and applied RFR model has been capable of utilizing both spatial and spectral information sources. In fact, the most important features identified by the algorithm above the threshold level of 0.05 (Section 3.2) are latitude, longitude, Rrs 708 and Rrs 778 nm, Rrs 400 nm, and MBR. Latitude and longitude indirectly influence TRIX values by mimicking the regional environmental conditions. The climate and related factors like temperature and precipitation, which, in turn, affect algal growth and nutrient levels, are affected by latitude. Longitude is partly related to the distance from the coast: in continental regions, the distance from the coast is related to nutrient concentrations based on land use and coastal dynamics. Insular regions, instead, are not affected by the same drivers. In fact, they show the lowest TRIX values in this study.

Following latitude and longitude, the RFR algorithm identified features connected to Chl-a retrieval: Rrs 708 and Rrs 778 nm, Rrs 400 nm, and MBR. This might be explained by the fact that these bands are used for the satellite retrieval of chlorophyll. In particular, Rrs 708 and Rrs 778 nm are within the NIR and red spectral bands, which are used for Chl-a estimation in coastal waters because they are less prone to interference from suspended sediments and CDOM. Their longer wavelengths and reduced sensitivity to scattering and absorption by these substances make them valuable for remote sensing in environments with variable water quality, such as coastal and nearshore areas [42]. The relevance of the Rrs 400 nm band, as it has its primary applications in assessing water quality and environmental parameters, is also sometimes considered in Chl-a retrieval algorithms for coastal waters [51]. However, the 400 nm band is typically secondary to the primary Chl-a-sensitive bands, like the blue and red bands, in these algorithms. Lastly, the feature above the threshold level of 0.05 (Section 3.2) is the MBR, a parameter that accounts for the primary absorption bands of Chl-a [52].

### 4.4. TRIX Estimates Obtained

Based on the aforementioned strengths, our investigation has succeeded in predicting the TRIX levels of the examined coastal waters with a moderate level of accuracy. The TRIX estimates obtained show space and time patterns which are generally explainable through basic ecological considerations. The highest TRIX values found near the estuaries of the main rivers, for example, are driven by several interconnected factors. This is the case for the Tiber River, which is a significant freshwater source delivering nutrients and organic matter into the coastal zone. Urbanization and agriculture in the Latium region introduce pollutants and nutrients via river runoff. The shallowness of coastal waters allows for efficient nutrient uptake by phytoplankton and the formation of algal blooms. Limited water exchange between the estuary and the open sea can trap and accumulate nutrients. The interplay of these factors results in eutrophic conditions.

The oligotrophic nature of Sardinia’s coastal waters can instead be attributed to limited nutrient inputs, geographical isolation, and relatively deep waters close to shore. The region experiences low levels of external nutrient sources, resulting in reduced nutrient availability for primary production. Clear waters and the absence of excessive phytoplankton growth are indicative of this oligotrophic state. Additionally, the island’s relatively low population density and limited industrial activity contribute to lower anthropogenic nutrient inputs, reinforcing its oligotrophic coastal conditions. More generally, the western seas in Italy, included in the study area, are known to exhibit increasing levels of phytoplankton biomass and primary production from south to north, thereby indicating a northward decreasing trend in oligotrophic characteristics. This trend is confirmed by the positive and significant correlation which was found between TRIX and latitude.

## 5. Conclusions

The TRIX is suitable for monitoring the ecological quality of coastal seawaters, which is important for both scientific and practical purposes. Both theoretical considerations and previous investigations indicate that the TRIX is estimable through the use of multi/hyperspectral remotely sensed data.

This subject was investigated by the application of an advanced machine learning algorithm, the RFR, to in situ TRIX observations and optical imagery collected by the OLCI sensor. The results obtained are encouraging, particularly for future operational monitoring activities. Our research, in fact, contributes to the advancement of TRIX assessment in challenging coastal environments, further highlighting the potential of remote sensing and machine learning in environmental monitoring and management.

On the one hand, the availability of a limited dataset of only 340 in situ TRIX matchups impacts the general validity of the quantitative errors shown, and, on the other hand, it leaves room for improvement with an increase in available measurements. The research, in fact, has identified several critical issues that require further investigation. This should concern both the methodologies applied, with the consideration of other TRIX retrieval algorithms, and the observations analyzed, with the use of additional in situ and satellite datasets.

The main scientific contributions brought by the current investigation can, therefore, be summarized as follows:

1. Optically complex water quality assessment by satellite: the research has addressed the challenge of evaluating coastal water quality using remote sensing OLCI Sentinel-3 data (unprecedently used for this application), providing a valuable methodology for monitoring and managing these complex environments.
2. Machine learning for TRIX estimation using Sentinel-3 OLCI Data: this paper has used machine learning techniques, specifically the RFR applied in a pioneering way to high spatial and spectral resolution Sentinel-3 OLCI data, to estimate the TRIX, a crucial indicator of ecological quality. This approach offers a novel way, which is efficient and cost-effective, to assess the water quality in coastal ecosystems.
3. Advancement on TRIX assessment: the paper advances the field by demonstrating the feasibility of estimating the TRIX using remotely sensed data.

Though we recognize the limitations of the available in situ dataset presented and emphasize the need for further investigation, this study opens the avenue for future advancements in satellite sensors in conjunction with machine learning applicability for coastal ecosystems and water quality assessment and management.

## Figures and Tables

**Table 1:** Average (AV) and standard deviation (STD) of the four seawater parameters contributing to TRIX in the selected database.

In Situ Data	AV	STD
Chl-a (µg/L)	0.23	0.35
aDO%	5.50	5.71
Total N (µg/L)	165.33	169.28
Total P (µg/L)	19.63	33.81

**Table 2:** Set of hyperparameters investigated to be used in RFR. The best set of hyperparameters resulting from RMS minimization is shown in bold.

***n*_estimator**	1	5	10	50	**100**	200	300
**max_depth**	5	**10**	unlimited	-	-	-	-
**max_features**	1	5	10	**15**	17	20	-

**Table 3:** Statistics referred to the scatter plot in Figure 5 (** = highly significant correlation, *p* < 0.01).

R^2^	RMSE	MAE
0.37 **	0.64	0.51

## Data Availability

The data cited in this manuscript are available in registries that are freely accessible to the public (https://www.isprambiente.gov.it/it; https://www.copernicus.eu/en, accessed on 2 May 2023). All the data involved in this study are reflected in the relevant figures and tables, and there are no additional data to be provided.
